# Evaluating the Effectiveness of the Flipped Classroom Model in Undergraduate Medical Education: A Quasi-experimental Study

**DOI:** 10.7759/cureus.86312

**Published:** 2025-06-18

**Authors:** Sibabratta Patnaik, Manas Ranjan Behera, Lipilekha Patnaik

**Affiliations:** 1 Pediatrics, Kalinga Institute of Medical Sciences, Bhubaneswar, IND; 2 Community Medicine, Institute of Medical Sciences & SUM Hospital, Bhubaneswar, IND

**Keywords:** active learning, education, faculty training, flipped class, flipped classroom, student engagement, students, teaching

## Abstract

Background: The flipped classroom (FC) model has gained increasing recognition as an innovative teaching strategy in medical education, addressing the limitations of passive learning in traditional methods. It takes learning outside the classroom and allows students to preview new material, while leaving classroom time for content-related discussions and joint tasks. However, limited studies have evaluated its impact in Eastern India, particularly in pediatric teaching. The study was conducted to evaluate the effectiveness of the FC model versus the traditional method of teaching in improving knowledge retention and engagement among undergraduate medical students studying pediatric respiratory illnesses.

Materials and methods: A prospective quasi-experimental study was conducted over six months (November 2021 to April 2022) among 130 eighth-semester medical students at Kalinga Institute of Medical Sciences (KIMS), Bhubaneswar. Participants were divided into two groups: one receiving traditional lectures and the other exposed to FC sessions covering pediatric respiratory competencies. Pre- and post-test scores were compared using SPSS (IBM Corp., Armonk, NY) with a significance threshold of P < 0.05. The scores were compared, and their perception toward the teaching tool was assessed by a questionnaire-based survey.

Results: A total of 130 students, 67 in the control group and 63 in the intervention group, were analyzed for their knowledge score at baseline by a pretested questionnaire. The mean score of knowledge among the control and intervention groups was not different at baseline (P = 0.519). Students in the FC group demonstrated a significantly greater improvement in knowledge scores (mean increase: 2.0 points) compared to the traditional group (mean increase: 0.925 points; P < 0.01). Additionally, 77.8% of participants reported increased engagement in the flipped sessions.

Conclusions: The greater mean scores after the teaching sessions represent the improved perception of the FC teaching-learning approach for repeatability and better memory retention. The FC method was found to be more effective than the traditional method of teaching. The students’ feedback was positive toward the FC method. With careful preparation and faculty training, it can address the issue of medical students' lack of competency in pediatrics.

## Introduction

The flipped classroom (FC) has been widely adopted across various countries and is acknowledged as an innovative and significant teaching strategy in higher education [1]. Education institutes are encouraged to develop the FC method further to enhance the classroom dynamics and foster a positive learning atmosphere [2,3]. It shifts learning beyond the classroom, enabling the students to preview new material while reserving class time for content-related activities and collaborative tasks [4-7]. This innovative approach enables educators to offer one-on-one instruction and fosters students' sense of responsibility for learning. Consequently, the FC model can enhance teaching by allowing the learners to apply and integrate the taught contents and even learning to evaluate and create [8].

For hundreds of years, medical content has been delivered in a mostly traditional style, with an instructor lecturing and a learner passively acquiring the information, which is then reviewed later when examinations approach. In the FC model, what is normally done in class is flipped or switched with what is normally done by the students out of class. This is done by completing some pre-class assignments, such as watching a short video, listening to a talk or presentation, or reading an article from a journal (self-studying the lecture or theory material) [1,3]. The class time is then dedicated to reinforcing and applying this knowledge through discussions, interactive exercises, group learning, and case studies [4].

In a study by Bhavsar et al., a significant difference (P < 0.05) was observed in post-test scores of both FC groups in both modules, and positive feedback was received for the FC method of teaching compared to the traditional method. Considering responses and results of the assessment, the authors concluded that the FC approach is beneficial for students [9].

The FC model has gained increasing recognition as an innovative teaching strategy in medical education, addressing the limitations of passive learning in traditional methods. However, limited studies have evaluated its impact in Eastern India, particularly in pediatric teaching. Therefore, this study aims to compare the effectiveness of the FC model versus traditional methods in improving knowledge retention, student engagement, and perceptions in undergraduate medical education.

## Materials and methods

Study design and patient selection

This was a prospective quasi-experimental study of a non-randomized control group design conducted over a period of six months, starting from November 2021 to April 2022. The Institutional Ethics Committee approval was taken prior to the study from the Institutional Ethics Committee, Kalinga Institute of Medical Sciences (KIMS), Bhubaneswar (Approval No.: KIIT/KIMS/IEC/540/2021).

All 8th-semester students of KIMS, Bhubaneswar, who gave informed consent (130 students) were included in this study (universal sampling). The participants were clearly informed about the purpose of the project, and written consent was obtained. Confidentiality was ensured at all stages. Participants were informed regarding the right to discontinue at any time without giving reasons. Participants were assigned alternately to the control and intervention group as per their roll number. The first roll number was assigned to the intervention group by the lottery method.

Data collection

The study tool was developed with departmental faculty consensus and validated by seven faculty members other than the study institute. They were requested to assess each questionnaire item in terms of relevance, clarity, simplicity, and ambiguity on a four-point scale. Content validity index (CVI) was evaluated. Experts rated each item on a Likert scale (1 = not relevant; 4 = highly relevant).

Two groups were formed with 67 and 63 students, and one teacher took a class for each group. One group had traditional teaching, and the other had a flipped class (four classes in four weeks for each group). The teacher who took the FC session was trained in an advanced course in medical education (ACME). The sessions covered the pediatrics competencies PE 27.3, 27.4, and 27.5, focusing on pediatric respiratory distress and pediatric shock. They included a comprehensive discussion on the clinical features, diagnosis, and management of conditions like bronchiolitis, croup, asthma, and pediatric shock, emphasizing evidence-based practices. The pre-test was done on the first day before starting classes with a predesigned and validated questionnaire. Post-test was done after the completion of four weeks. The pre-class materials included a few journal articles and short videos on the topic of discussion. During FC sessions, interactive classes were undertaken along with PowerPoint (Microsoft Corporation, Redmond, WA) presentations. In the traditional group, a PowerPoint presentation was used along with interactive classes. The traditional group was taught again by the flipped classroom method for their benefit, if any. After the classes were finished, students’ perception was assessed by a questionnaire-based survey.

A feedback form has been developed to collect feedback from students regarding flipped classroom teaching. The feedback form was filled up by the students of the intervention and control groups using a Likert scale. The scale assumed the strength of an attitude of students through a linear form, like strongly agree, agree, neutral, disagree, and strongly disagree. The feedback form included seven questions, including interest, understanding, motivation, and usability of flipped classroom teaching.

Outcome measures

The primary outcome was the knowledge score of the students. The knowledge score was calculated from a predesigned and validated questionnaire. The feedback form included seven questions, including interest, understanding, motivation, and usability of flipped classroom teaching. The feedback form was filled up by the students using a Likert scale through a linear form like strongly agree, agree, neutral, disagree, and strongly disagree.

Statistical analysis

The results were analyzed and compared. The data collected were entered in a Microsoft Excel spreadsheet (Microsoft Corporation) and imported to SPSS for Windows version 20.0 (IBM Corp., Armonk, NY). The data were analyzed using SPSS. Descriptive statistics were expressed as frequencies (percentages), means, medians, standard deviations, and 95% confidence intervals. The missing data, i.e., data from students who were absent in the post-test and students who had given the post-test without the pre-test, were excluded from the analysis. The effect size (Cohen’s d) was calculated ((8.05-6.76)/2.07 = 0.62), which was a moderate effect size. This suggests that the difference is noticeable and potentially meaningful in a real-world context. To compare the means of continuous variables, an independent t-test was used. A paired t-test was done for the change in knowledge score after intervention in each group. Feedback was presented as percentages. To examine the association between categorical variables, the chi-square test was used. Statistical significance was defined as a p-value of 0.05 or less.

## Results

A total of 130 students, 67 in the control group and 63 in the intervention group, were analyzed for their knowledge score at baseline by a predesigned and validated questionnaire. The mean score of knowledge among the control and intervention groups was 5.84 ­± 2.04 and 6.05 ± 1.68, respectively, at baseline. The mean score of knowledge between the control and intervention groups was not different at baseline (P = 0.519).

The knowledge score increased to 6.76 ± 2.13 from 5.84 ­± 2.04 in the control group (P < 0.01) and 8.05 ± 2.01 from 6.05 ± 1.68 in the intervention group (P < 0.01). The difference in knowledge score (post-test minus pre-test) was increased by 0.925 points in the control group and by two points in the intervention group. This change was significantly higher in the flipped classroom group (P < 0.01) (Table 1).

**Table 1:** Change in knowledge scores.

Knowledge score	Baseline (pretest) (Mean ± SD)	Follow-up (post-test) (Mean ± SD)	Difference (Follow-up –baseline)	P-value (paired T-test)
Control (n = 67)	5.84 ­± 2.04	6.76 ± 2.13	0.925 (0.47-1.37)	<0.01
Intervention (flipped) (n = 63)	6.05 ± 1.68	8.05 ± 2.01	2.0 (1.41-2.58)	<0.01

**Figure 1:**
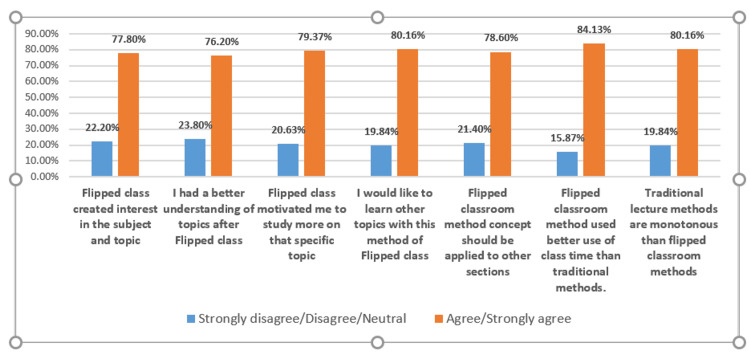
Feedback of students on the flipped classroom method.

Feedback was collected from all 130 students, and the response rate was 100%. Positive feedback was given by around 80% of students. More than 75% of students agreed that the flipped classroom session created interest among them, they became motivated toward the subject, and they had a better understanding of the topic. Around 80% of students were interested to learn other topics by the flipped classroom method. More than 80% of students agreed that the sessions were monotonous due to traditional teaching. Overall, around 80% of students gave positive feedback on the FC method of teaching (Figure 1).

## Discussion

In our study, the flipped classroom method significantly improved students’ knowledge scores compared to traditional teaching. Additionally, over 80% of students reported increased interest, motivation, and understanding of the subject, expressing a preference for this method over traditional lectures.

The flipped classroom essentially consists of three basic components: pre-class, in-class, and post-class assignments. The most important challenge for the facilitator is to ensure compliance with the pre-class material. It was noted that suboptimal student preparation and insufficient direction may limit the student-centered benefits [10]. The FC model was found to be very effective, and the students’ feedback was very encouraging, making it very relevant in medical education.

In our study, nine students (15%) came without going through the pre-class module. It was demonstrated in a previous study that if students came prepared, they performed better [11]. This study was conducted during the COVID-19 pandemic; thus, the classes were taken online. A similar study was published recently by Binnie and Bonsor in the British Dental Journal, where they found success in the flipped model of teaching during COVID-19 [12]. Our results were almost similar, where more than 80% of students were satisfied.

Flipping the class addressed the challenges of limited contact time for a time-restricted lecturer and allowed exploration of a novel teaching method. This change fostered more dynamic interaction, making a positive learning environment for both teachers and students.

In a meta-analysis, it was found that the use of quizzes at the start of class would make the flipped classroom more effective [13]. We have not tried this, but it seems to be feasible and can be applied. It is believed that by quizzes, the students can recall the subject they had learnt prior to the class.

FC model’s focus on pre-class preparation allows students to engage in higher-order cognitive skills during class, such as application and evaluation, which aligns with Bloom’s taxonomy.

A few students had commented that it took a lot of time to study the pre-course material and watch videos. It is a practical problem found in many studies, and it can be suggested that the FC approach may be implemented only for difficult and complex topics, which need repeated reading, while other topics can be taught in traditional methods. This approach can save time for students. In addition, one can limit the duration of video segments to 20 minutes. Many studies, though non-health related, have promoted this [14,15]. Chuang et al. suggested that this approach is suitable for those learners who are motivated and hardworking [16]. Still, around 26 students (20%) in our study did not like the FC method. General thinking is that the learners from professional courses will like the FC method, but it may not always be true. Patanwala et al. found this in students from a pharmacy institute, who did not come prepared despite advice [17]. This indirectly shows the role of a facilitator in stimulating the learners to come prepared and to adapt to the method.

In a recent study from the United States, students liked the FC method, and there was an improvement in the knowledge of students; however, the preparatory time of students was increased [18]. Factors such as interactive activities, peer collaboration, or instructor involvement may have contributed to the improved engagement in our study.

Some studies have reported minimal benefits from FC due to differences in implementation strategies, student preparedness, or content complexity.

Providing specific suggestions, such as reducing pre-class video durations, incorporating brief assessments to reinforce learning, or offering faculty training to ensure consistent delivery, would make the discussion more impactful for practitioners. FC model’s focus on pre-class preparation allows students to engage in higher-order cognitive skills during class, such as application and evaluation, which aligns with Bloom’s taxonomy.

One of the major challenges is that the teacher or guide is the main person for the success of the FC approach. The knowledge of the teacher should be significantly high level, enabling them to steer discussions in any direction and explore topics in sufficient depth. The study was conducted at a single institution, and they failed to discuss other potential concerns such as sample size, instructor bias, or the short duration of the intervention. The students take ownership of their learning, but the facilitator must be able to respond and accommodate. A lot of time is to be spared by the teacher to prepare the pre-course material, including videos and podcasts. Additionally, they must be proficient in software and editing skills [12]. Despite these challenges, it is an excellent innovative teaching method, which can be recommended in medical teaching.

The limitation of the study is that it is from one department, and the study participants belong to one institute. The duration of the intervention was also very short. Instructor bias is also a limitation of this study. The long-term impact of the FC method and whether the students can retain the knowledge for a prolonged period is not assessed in our study. Involving multiple centers and including many departments can be a better option.

## Conclusions

The flipped classroom method was found to be more effective than the traditional method of teaching. The FC model has the potential to improve medical education outcomes. The students’ feedback was positive toward the flipped method. The greater mean scores after the teaching sessions represent the improved perception of the flipped classroom teaching-learning approach for repeatability and better memory retention. With careful preparation and faculty training, it can address the issue of medical students' lack of competency in pediatrics up to the level of "know how." Since FC sessions promote self-directed learning and case-based learning, this innovative teaching strategy can be employed for medical students.

## Appendices

Questionnaire

1. A one-year-old boy presented with a cough and cold for three days. He had a mild fever. On examination, his heart rate (HR) was 100, respiratory rate (RR) was 40, oxygen saturation (SPO2) was 95%, and temperature was 100°F. There was stridor present. What is correct about the patient?

1. He is in respiratory distress, and the cause is upper airway obstruction

2. He is in respiratory failure

3. He requires urgent oxygen therapy for the treatment of pneumonia

4. Steroids are urgently required

2. A six-month-old, previously healthy baby presenting with mild fever, cold, and rapid breathing for two days. On examination, HR was 149, RR was 64, temperature was normal, and SPO2 was 80% in room air. Bilateral diffuse wheeze was present. Mild chest retractions were present.

1. The child needs urgent intubation and ventilator treatment because of respiratory failure

2. Moist oxygen and normal saline nebulization will NOT be helpful

3. The child is in respiratory distress, and it is due to lower airway obstruction

4. Oxygen by mask will be better than high-flow nasal cannula (HFNC).

3. The minimum amount of oxygen provided by the Hudson simple face mask is

1. 10 L

2. 8 L

3. 4 L

4. 2 L

4. Which of the following about the oxygen dissociation curve is incorrect?

1. Raised temperature shifts it to the right

2. Abnormal hemoglobin (Hb) can shift it to the left

3. Acidosis can shift it to the left

4. 2,3-diphosphoglyceric acid (2,3-DPG) can shift it to the right

5. Which of the following about oxygen delivery systems is correct?

1. A partial rebreathing mask delivers more oxygen than a non-rebreathing mask

2. Venturi is a low-flow device

3. Nasal prong can be high flow for a newborn baby

4. Hood box can use as low as 1 L of oxygen flow

6. A 10-year-old boy presented to the emergency department with a history of fever and headache for two days and unconsciousness for the last six hours. There were a lot of secretions in the mouth. SPO2 was 70% in room air, and it increased to 90% with a non-rebreathing mask at 10 L. Respiratory rate was 8-10/min. Bilateral vesicular breath sounds with conducted sounds were noted. Deep jerks are brisk, and pupils are constricted bilaterally. What needs to be done now?

1. Urgent neurology consultation to see for cause of unconsciousness

2. Urgent intubation and ventilation for respiratory failure

3. Mannitol is urgently needed to control intracranial pressure

4. Lumbar puncture is a must in the ED to rule out meningitis

7. A two-month-old baby presented with excessive coughing and mild fever. Since morning, she has not been breastfeeding and is very cranky. On examination, HR was 180, RR was 80, retractions ++, and bilateral diffuse wheeze was present. SPO2 was 80%, which improved to 92% after nebulization with saline and oxygen 2 L/min by nasal prongs. Which cannot be a possibility in this case?

1. Respiratory failure can NOT lead to cardio-respiratory failure

2. She may improve with treatment with oxygen only

3. Some may need intubation and ventilation

4. Proper monitoring and timely intervention are important

8. How much fraction of inspired oxygen (FIO2) can be generated by a simple face mask?

1. Maximum 45-50%

2. 80%

3. 21%

4. 60%

9. Which of the following is a low-flow device?

1. Simple face mask

2. Non-rebreather mask (NRM)

3. HFNC

4. Venturi

10. Which of the following is a sign of respiratory failure in a one-year-old child with pneumonia?

1. Severe retraction

2. Respiratory rate of 14/min

3. SPO2 of 92% with face mask

4. Bilateral diffuse wheeze and crackles

11. In an acute severe asthma child, which of the following depicts respiratory failure?

1. Diffuse wheeze with retraction

2. SPO2 of 90% with NRM and partial pressure of carbon dioxide (PCO2) of 60 in arterial blood gas (ABG)

3. Unconscious child

4. No response to salbutamol nebulization

12. Management of anaphylaxis is by

1. Inj. Adrenalin (1:10000) 0.1 ml/kg IV

2. Inj. Adrenalin (1:1000) 0.1ml/kg IV

3. Inj. Adrenalin (1:1000) 0.01ml/kg IM

4. Inj. Hydrocortisone 2mg/kg IV

13. Which of the following about the O2 delivery system is incorrect?

1. NRM is good for newborns

2. HFNC can be used in infants and adults

3. A face mask does not require proper sealing

4. NRM does require a proper seal

14. A two-year-old child having a fever for five days, high grade with coughing and tachypnea. X-ray revealed bilateral haziness. SPO2 was 60% in room air and 95% with HFNC. Bilateral diffuse crackles present. Neutrophilia with high CRP is present. Which of the following is incorrect?

1. Antibiotics are a must

2. May require ventilation

3. High flow is helpful

4. Nasal cannula is best
